# Polymer-Supported Raney Nickel Catalysts for Sustainable Reduction Reactions

**DOI:** 10.3390/molecules21070833

**Published:** 2016-06-25

**Authors:** Haibin Jiang, Shuliang Lu, Xiaohong Zhang, Wei Dai, Jinliang Qiao

**Affiliations:** SINOPEC Beijing Research Institute of Chemical Industry, Beijing 100013, China; jianghb.bjhy@sinopec.com (H.J.); lusl.bjhy@sinopec.com (S.L.); zhangxh.bjhy@sinopec.com (X.Z.); daiw.bjhy@sinopec.com (W.D.)

**Keywords:** polymer-supported, Raney catalysts, green chemistry

## Abstract

Green is the future of chemistry. Catalysts with high selectivity are the key to green chemistry. Polymer-supported Raney catalysts have been found to have outstanding performance in the clean preparation of some chemicals. For example, a polyamide 6-supported Raney nickel catalyst provided a 100.0% conversion of *n*-butyraldehyde without producing any detectable *n*-butyl ether, the main byproduct in industry, and eliminated the two main byproducts (isopropyl ether and methyl-*iso*-butylcarbinol) in the hydrogenation of acetone to isopropanol. Meanwhile, a model for how the polymer support brought about the elimination of byproducts is proposed and confirmed. In this account the preparation and applications of polymer-supported Raney catalysts along with the corresponding models will be reviewed.

## 1. Introduction

Catalytic reactions frequently bring about side reactions. In the chemical industry these side reactions, consuming a large amount of resources and energy and producing a mass of waste, cause seriously negative impacts on the environment. In the pharmaceutical and food industry impurities could harm and even threaten our life. Therefore, it is of great significance to improve catalytic selectivity to reduce and if possible eliminate side reactions.

Raney catalysts (e.g., Raney nickel, Raney cobalt, and Raney copper) are a series of important catalysts routinely used in the chemical industry. Raney catalysts, with their high specific surface area, offer the advantage over conventionally supported metal catalysts of having a high catalytic activity at a relatively low temperature. Nevertheless, Raney catalysts have some disadvantages as well, which limit their applications. For example, powdered Raney catalysts cannot be applied in fix-bed reactors. Therefore, currently they are mainly employed in slurry phase reactors for small batch production. In this case, catalytic selectivity is hard to control [1]; moreover, Raney catalysts must be separated from the reaction medium. Another shortcoming of Raney catalysts is that inevitably they contain a certain fraction of Al_2_O_3_ [2], and the acidity of Al_2_O_3_ often leads to some side reactions. In order to overcome the disadvantages of Raney catalysts, a number of researchers have devoted their efforts to shaping Raney catalysts for fixed-bed reactions [3,4,5,6,7,8,9,10,11,12,13,14,15,16,17], which, however, didn’t work out satisfactorily [18].

Polymer materials with different structures, possessing excellent processibility, recyclability and surface properties, could be ideal alternatives for replacing conventional catalyst supports to meet different specific demands of catalysts for different chemical reactions. More importantly, the acidity or alkalinity of polymer materials could be adjusted at the molecular level by means of chemical functionalization, so that the corresponding side reactions could be minimized. As such, polymers could be an optimum kind of catalyst support for green chemistry as long as the specific surface area of the final catalyst could be large enough, which makes porous Raney metals suitable components for polymer-supported catalysts.

Combining the merits of both Raney metals and polymers, polymer-supported Raney catalysts with high selectivity were developed for fixed-bed reactions [19], and have been successfully applied to many chemical reactions [18,20,21,22,23,24,25,26,27,28,29,30,31,32,33,34]. The major disadvantages of Raney catalysts were satisfactorily overcome. Moreover, the preparation and recycle processes of these catalysts are much more eco-friendly than that of those traditional Al_2_O_3_-/SiO_2_-supported catalysts [19]. In this account, the preparation and applications along with corresponding models of polymer-supported Raney catalysts will be reviewed.

## 2. Preparation

**Figure 1:**
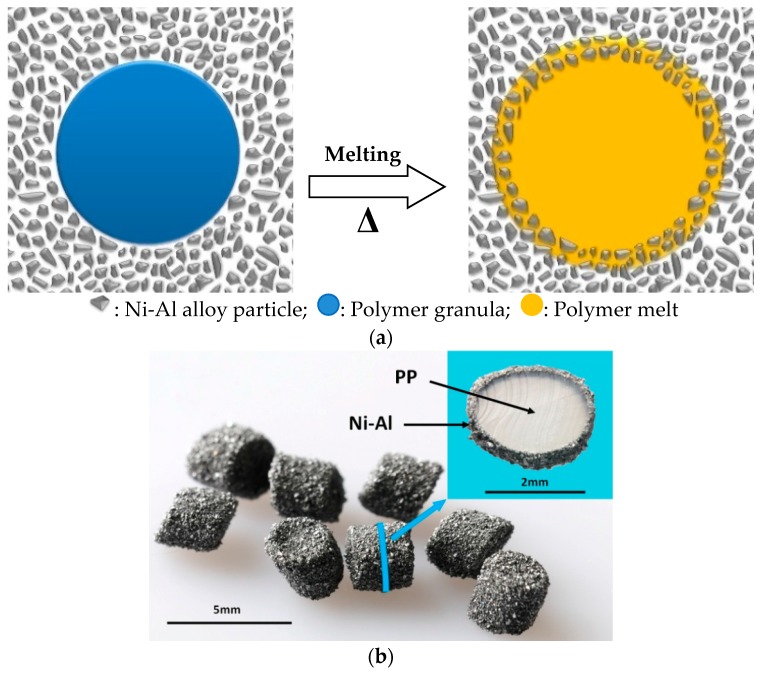
(**a**) Schematic representation of the process whereby Raney alloy particles are embedded in a polymer surface; (**b**) Photograph of the special granules. Inset shows the sectional view of a cut sample [19]. Reproduced from Ref. [19] with permission from the Royal Society of Chemistry.

Polymer-supported Raney catalysts are typically prepared as follows [19,21]: polymer granules are separately buried into a full mold of Raney alloy powder at the temperature 30–50 °C higher than melting point of the polymer used, and then the mold is compressed (2 MPa). Thus, Raney alloy powders are embedded into the surface of the polymer granules. Thereafter, the mold is cooled down to give special granules, on which surface the Raney alloy particles are embedded as shown in Figure 1. After sieving out the special granules from the excess Raney alloy powder, the polymer-supported Raney catalyst is obtained after alkaline leaching of these special granules.

## 3. Application of Polymer-Supported Raney Catalysts in Clean Preparation of *n*-Butanol

We [19,21,22,30] firstly prepared three different catalysts for the hydrogenation reaction of *n*-butyraldehyde, which were neutral polypropylene (PP)-supported Raney Ni catalyst (Raney Ni/PP), acidic maleic anhydride grafted PP (MAHPP)-supported Raney Ni catalyst (Raney Ni/MAHPP), and traditional Al_2_O_3_-supported Ni catalyst (Ni/Al_2_O_3_). This hydrogenation reaction has one main side reaction, which is acid-catalyzed, and the yield of the *n*-butyl ether byproduct increases with increasing acid strength of the catalyst [35,36]. The worldwide consumption of *n*-butanol is more than 3 million tons per year, and in order to separate *n*-butyl ether from *n*-butanol, a large amount of energy is required because an azeotrope is formed. For the reduction of pollution, and energy and resource consumption in *n*-butanol production, it is important to eliminate this side reaction. We found, as shown in Table 1, that the neutral support PP effectively reduced the side reaction with respect to those acidic supports (i.e., MAHPP and Al_2_O_3_). However, although the fraction of the byproduct was very small, the Raney Ni/PP catalyst didn’t eliminate the *n*-butyl ether byproduct completely because of the residual Al_2_O_3_ in the Raney Ni [2]. In order to further reduce the acid-catalyzed side reaction, we then prepared alkalescent polyamide 6 (PA6)-supported Raney Ni catalyst (Raney Ni/PA), PA6 with lone pair electrons at the N atom for every repeating unit. Significantly, a clean preparation of *n*-butanol with a 100% conversion and undetectable *n*-butyl ether byproduct was achieved with the Raney Ni/PA catalyst at a relatively low temperature (110 °C, also see Table 1).

**Figure 2:**
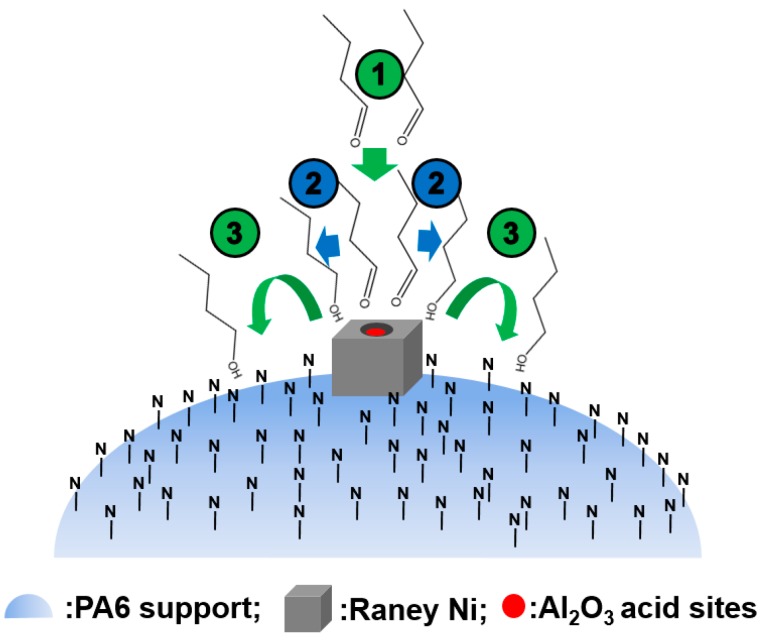
Schematic representation of elimination of *n*-butyl ether over Raney Ni/PA catalyst.

In order to reveal how the PA6 support could diminish the side reaction brought about by the Al_2_O_3_ in Raney Ni, we [19] firstly confirmed, by XPS measurements, that the basic N atom in PA6 did not affect the acidity of Al atom of Al_2_O_3_ in the Raney Ni because of the relatively large “intermolecular distance”. The XPS Al 2s peaks of the Raney Ni/PA and Ni/Al_2_O_3_ catalysts were located at 74.03 and 73.93 eV, respectively, which were almost the same within the experimental error and indicated no charge-transfer (base-acid neutralization) interaction between basic PA6 and acidic Al_2_O_3_ in the Raney Ni. It is well accepted that the adsorption ability of a catalyst support to reactants and products can largely affect the catalytic reactivity [37,38]. Since the N atoms in PA6 can form hydrogen bonds with the -OH groups in *n*-butanol and the interval between every two neighboring N atoms in PA6 molecule chain is only about 0.86 nm, the PA6 support possesses strong adsorption ability toward *n*-butanol. Therefore, we considered that the following processes might have occurred (see Figure 2). Process 1: *n*-butyraldehyde was adsorbed by the Raney Ni of the Raney Ni/PA catalyst; process 2: *n*-butyraldehyde was catalytically reduced to *n*-butanol by Ni metal; process 3: once produced, *n*-butanol was selectively adsorbed by N atoms in the PA6, rather than the acidic Al atoms in the Raney Ni. Unlike the acidic Al atoms in Al_2_O_3_, the basic N atoms in the PA6 support couldn’t catalyze *n*-butanol to *n*-butyl ether conversion. For the Raney Ni/MAHPP catalyst, the maleic anhydride in the MAHPP support could also selectively adsorb *n*-butanol over Al_2_O_3_. However, the acidic maleic anhydride could effectively catalyze the *n*-butanol to *n*-butyl ether step, leading to the formation of even more *n*-butyl ether by the Raney Ni/MAHPP catalyst with respect to the Ni/Al_2_O_3_ catalyst (see Table 1). Clearly, therefore, it was the interplay of the alkalinity and strong adsorption ability of *n*-butanol to intrinsically associate with the N atoms in the PA support that made the clean preparation of *n*-butanol by the Raney Ni/PA catalyst possible. The relationships between the alkalinity or acidity of the catalyst supports and the byproduct content (*n*-butyl ether) are summarized in Table 2.

**Figure 3:**
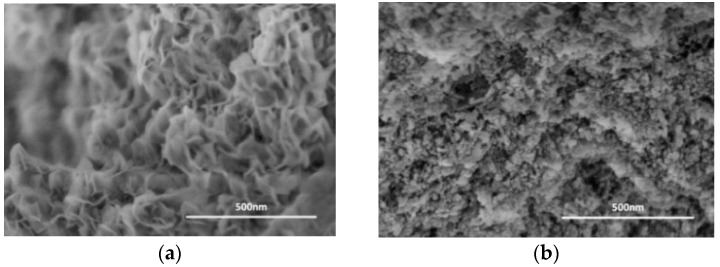
SEM images of (**a**) the Ni/PA catalyst and (**b**) the Ni/Al_2_O_3_ catalyst [19]. Reproduced from Ref. [19] with permission from the Royal Society of Chemistry.

To further illustrate the difference between the Raney Ni/PA catalyst and Ni/Al_2_O_3_ catalyst, we [19] performed SEM (see Figure 3) studies and found quite different surface morphologies and different porosity. Besides, based on the BET and XPS results, we found that even though the BET specific surface area of the Raney Ni/PA catalyst (only 4.5 m^2^/g) was much lower than that of Ni/Al_2_O_3_ catalyst (tens to hundreds m^2^/g), the Raney Ni/PA catalyst possessed higher catalytic activity owing to the surface of Raney Ni/PA catalyst mostly covered by active Ni component.

**Figure 4:**
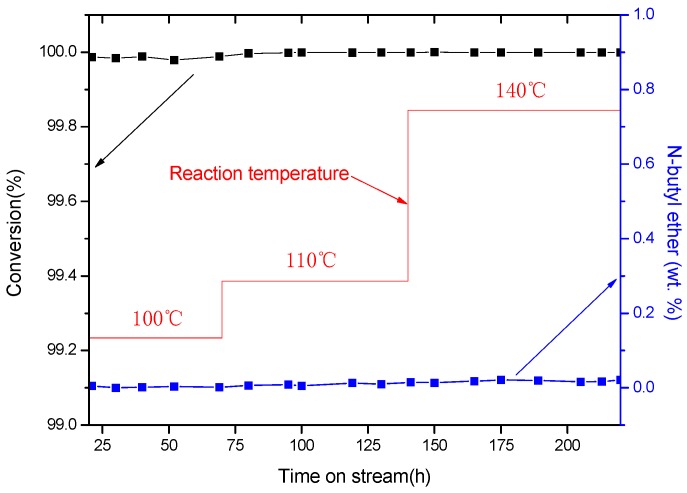
Long-term catalytic test for the Raney Ni/PA catalyst under a pressure of 4.0 MPa at different temperatures over 100–140 °C; red stepwise lines represent the temperature sequence used for the test [19]. Reproduced from Ref. [19] with permission from the Royal Society of Chemistry.

Finally, the long-term activity and selectivity of the Raney Ni/PA catalyst were also investigated (see Figure 4), and showed that the Raney Ni/PA catalyst had an excellent performance in terms of both activity and selectivity over long-term operation.

## 4. Application of Polymer-Supported Raney Catalysts in the Hydrogenation of Acetone to Isopropanol

Since hydrogenation reaction of *n*-butyraldehyde has only one main side reaction, in order to investigate the performance of polymer-supported Raney catalysts in a relatively more complex reaction system, we [18] prepared four different catalysts for the hydrogenation of acetone to isopropanol, which were Ni/Al_2_O_3_ catalyst, Al_2_O_3_-supported Raney Ni catalyst (Raney Ni/Al_2_O_3_), unsupported granular Raney Ni catalyst, and Raney Ni/PA catalyst. This hydrogenation reaction has two main byproducts, isopropyl ether and methyl-*iso*-butylcarbinol (MIBC) (see Reactions (1)–(4)) [39,40,41].


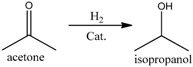
(1)


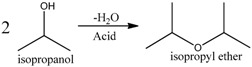
(2)


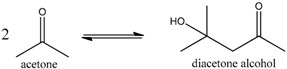
(3)


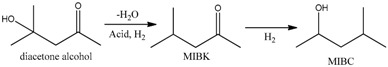
(4)

We found that the three Raney Ni-related catalysts all had both higher activity and selectivity than the Ni/Al_2_O_3_ catalyst (see Table 3), indicating Raney Ni’s good catalytic performance for the hydrogenation of acetone to isopropanol. That was because the acidity of Al_2_O_3_ could catalyze the generation of byproducts in this reaction and the Al_2_O_3_ in Ni/Al_2_O_3_ catalyst apparently had much more opportunities to catalyze the side reactions than the Al_2_O_3_ remaining inside the pores of Raney Ni resulting from the incomplete leaching of Al in Ni-Al alloy [2]. Moreover, supported by PA6, Raney Ni not only maintained its high activity, but even reached 100% selectivity to achieve clean preparation of isopropanol. The support-effect model proposed in clean preparation of *n*-butanol was also used to explain why both byproducts isopropyl ether and MIBC were eliminated.

**Figure 5:**
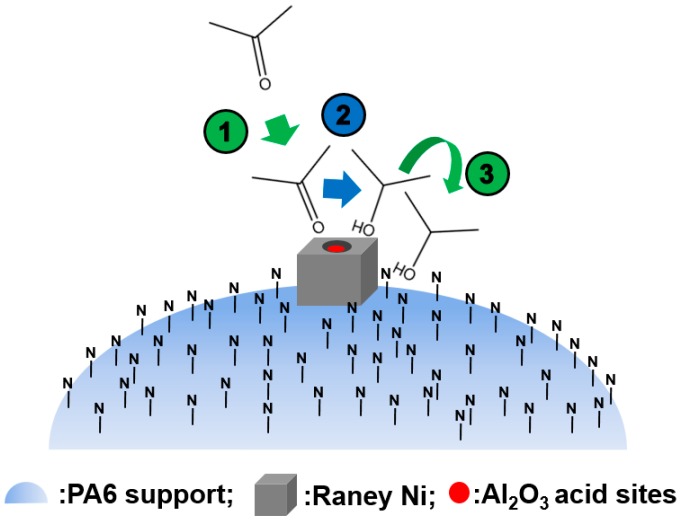
Schematic representation of the elimination of isopropyl ether over Raney Ni/PA catalyst. Reproduced from [18] with permission from Jiang H., Sci. China Chem.; published by Springer, 2016.

The isopropyl ether byproduct was generated from the main product isopropanol, catalyzed by acidic Al_2_O_3_ (Reaction (2)). Once adsorbed by the remaining Al_2_O_3_ inside the pores of Raney Ni, isopropanol would be catalytically converted to the byproduct isopropyl ether. As already noted, the PA6 support possesses strong adsorption ability for the -OH groups of isopropanol. Therefore, as shown in Figure 5, once produced, isopropanol was selectively adsorbed by N atoms in the PA6, rather than the acidic Al atoms inside the pores of Raney Ni. Unlike the acidic Al atoms in Al_2_O_3_, the basic N atoms in the PA6 support cannot catalyze the conversion of isopropanol to isopropyl ether. Clearly, it was also the interplay of the alkalinity and strong adsorption ability to isopropanol intrinsically associated with the N atoms in the PA6 support that made the elimination of isopropyl ether possible.

**Figure 6:**
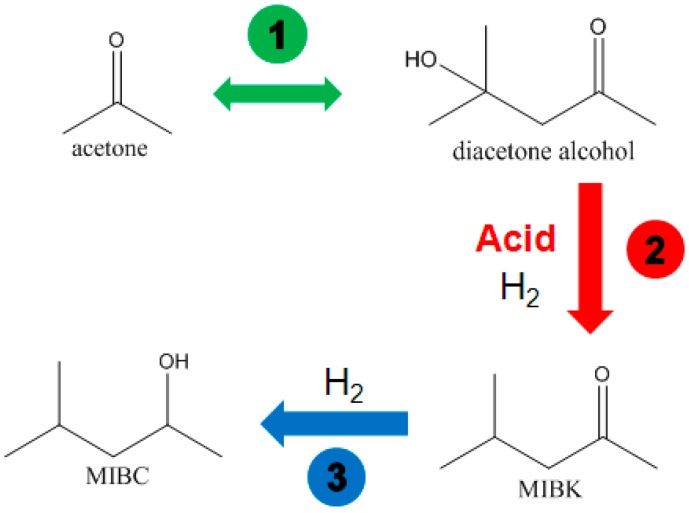
Schematic representation of generation process of byproduct MIBC. Reproduced from [18] with permission from Jiang H., Sci. China Chem.; published by Springer, 2016.

**Figure 7:**
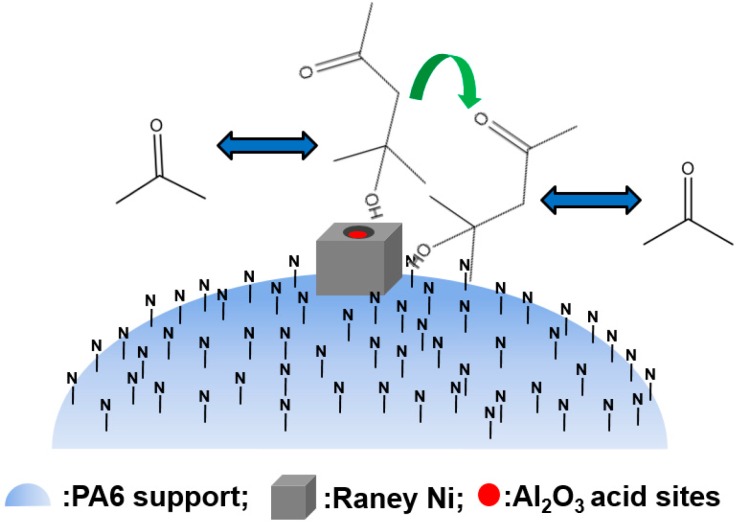
Schematic representation of elimination of MIBC over Raney Ni/PA catalyst. Reproduced from [18] with permission from Jiang H., Sci. China Chem.; published by Springer, 2016.

The byproduct MIBC was generated through the reaction processes shown in Figure 6 [39]. Obviously, removing the effect of acidic Al_2_O_3_ was also the key to eliminating the MIBC byproduct. The PA6 support possesses strong adsorption ability toward the -OH groups of diacetone alcohol; therefore, the byproduct MIBC was eliminated just like the byproduct isopropyl ether was, as shown in Figure 7.

## 5. Application of Polymer-Supported Raney Catalysts in Hydroamination of Acetone to Isopropylamine

It was obvious that the basic N atoms in the PA6 support having strong interactions with reactants plays a key role in the clean preparation of both *n*-butanol and isopropanol. In order to confirm the above conclusion, we studied the hydroamination of acetone to isopropylamine [18]. Similarly, this reaction has two main side reactions, which generate the byproducts diisopropylamine (DIPA) and isopropanol, respectively (see Reactions (5)–(7)). We found that conversion, selectivity, and byproduct contents over the four catalysts displayed a monotonically changing relationship (see Table 4). The support-effect model proposed in the clean preparation of *n*-butanol was again successfully used to explain the reaction results. NH_3_, easily adsorbed by Al_2_O_3_ acidic sites, was indispensable for the production of both the main product isopropylamine and the byproduct DIPA (see Reactions (5) and (6)). Therefore, Al_2_O_3_/Ni catalyst, with the highest Al_2_O_3_ content, possessed the highest activity and byproduct DIPA content among these four catalysts. Since there was just a small amount of Al_2_O_3_ remaining inside the pores of Raney Ni, the Al_2_O_3_ contents of these four catalysts showed the following ranking: Ni/Al_2_O_3_ > Raney Ni/Al_2_O_3_ > Granular Raney Ni > Raney Ni/PA, which was in agreement with that of conversion and byproduct DIPA content.


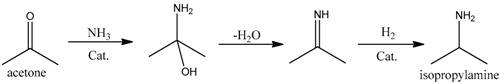
(5)


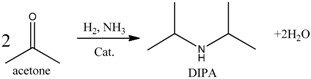
(6)


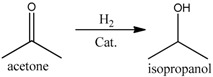
(7)

**Figure 8:**
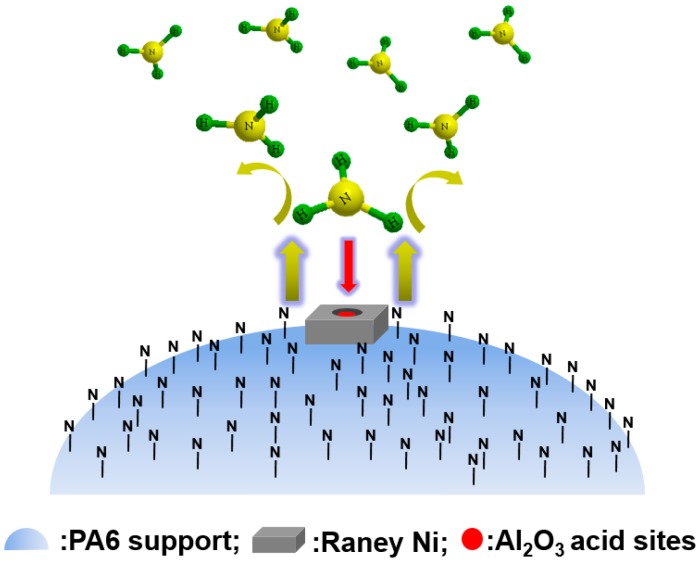
Schematic representation of prevention process of NH_3_ from adsorption. Reproduced from [18] with permission from Jiang H., Sci. China Chem.; published by Springer, 2016.

Furthermore, we also explain the Raney Ni/PA catalysts’ specificity, with very low activity and selectivity, but without any detectable DIPA byproduct. That was because the repulsive interaction between the N atoms in the PA6 support and NH_3_ is very strong. As shown in Figure 8, although the remaining Al_2_O_3_ inside the pores of Raney Ni could adsorb reactant NH_3_, the repulsive interaction of the PA6 support surrounding the Raney Ni prevented NH_3_ from being adsorbed by the Al_2_O_3_ inside the pores of Raney Ni. Therefore, the hydroamination reaction was difficult to conduct, or we can say there was very low activity and selectivity. Besides, as the very limited amount of adsorbed NH_3_ was exhausted by main Reaction (5), side Reaction (6) was eliminated. Moreover, the content of byproduct isopropanol even greatly exceeded that of main product isopropylamine over the Raney Ni/PA catalyst, because the generation of byproduct isopropanol did not need the presence of NH_3_.

It was clear and interesting that the catalyst support could play an important role in the chemical reactions. Different products could be produced when different catalyst supports were used, even when the chemicals, active component of the catalyst and reaction conditions were same. The main reactions and side reactions could even be reversed sometimes.

## 6. Application of Polymer-Supported Raney Catalysts in Other Chemical Reactions

Besides in reactions with selectivity issues, polymer-supported Raney catalysts were also successfully applied in other examples. Thanks to the much more eco-friendly preparation and recycle processes of polymer-supported Raney catalysts, it is meaningful to substitute them for traditional Al_2_O_3_-/SiO_2_-supported catalysts in some reactions. We have used the Raney Ni/PA catalyst to refine ethylene glycol (EG), with the ultraviolet (UV) transmittance of EG at wavelength of 220 nm, 275 nm, and 350 nm improved from 26.4%, 43.8% and 61.0% to 42.8%, 63.8% and 84.4%, respectively [31]. We also applied the Raney Ni/PA catalyst in low-temperature methanation reactions [25], and found that the Raney Ni/PA catalyst had higher catalytic activity than the current commercial catalyst under both normal and high pressures. Many other chemical reactions using polymer-supported Raney catalysts have been patented in China and other countries [20,21,23,24,26,27,28,29,32,33,34].

## 7. Outlook

High activity, high selectivity, good stability, and eco-friendly catalyst preparation and recycling processes make the polymer-supported catalysts promising for green chemistry. Though outstanding performances in many applications have been reported as summarized in this review, polymer-supported catalysts’ potential are far from being fully revealed. We believe that the catalytic selectivity of Raney catalysts and other hydrogenation catalysts in chemical industry, oil refining industry, pharmaceutical industry, and food industry, could be greatly improved by selecting appropriate polymer supports according to the features of specific reactions.

## Figures and Tables

**Table 1:** Hydrogenation of *n*-butyraldehyde with different catalysts over 100–140 °C [19]. Reproduced from Ref. [19] with permission from the Royal Society of Chemistry.

Catalyst	T (°C)	Conversion (%)	*n*-Butyl Ether (wt %)
Raney Ni/PP	100	99.99	0.013
	110	100	0.053
	120	100	0.095
	140	100	0.499
Raney Ni/MAHPP	100	99.99	0.300
	110	100	0.632
	120	100	1.049
	140	100	1.843
Ni/Al_2_O_3_	100	100	0.159
	110	100	0.292
	120	100	0.677
	140	100	1.706
Raney Ni/PA	100	99.99	undetected
	110	100	undetected
	120	100	0.015
	140	100	0.016

**Table 2:** The relationship between the property of catalyst support and the byproduct content [19]. Reproduced from Ref. [19] with permission from the Royal Society of Chemistry.

	Inorganic Support	Organic Support	Organic Support with Acid or Alkaline Group Which Can Adsorb *n*-Butanol	
**Support with alkalinity or acidity**	Al_2_O_3_ (acidity)	PP (neutral)	PP-g-MAH (acidity)	PA6 (alkalinity)
***n*-Butyl ether content**	High	Low	Very high	Very low to undetectable

**Table 3:** Hydrogenation of acetone with different catalysts at 132 °C [18]. Reproduced with permission from Jiang H., Sci. China Chem.; published by Springer, 2016.

Catalyst	Conversion (%)	Selectivity (%)		
		Isopropanol	Isopropyl Ether	MIBC
Ni/Al_2_O_3_	98.97	99.87	0.05	0.08
Raney Ni/Al_2_O_3_	99.71	99.91	0.04	0.05
Granular Raney Ni	99.76	99.94	0.04	0.02
Raney Ni/PA	99.75	100.00	undetectable	undetectable

**Table 4:** Hydroamination of acetone with different catalysts over 153°C. Reproduced from [18] with permission from Jiang H., Sci. China Chem.; published by Springer, 2016.

Catalyst	Conversion (%)	Selectivity (%)		
		Isopropylamine	DIPA	Isopropanol
Ni/Al_2_O_3_	99.38	79.06	7.00	13.94
Raney Ni/Al_2_O_3_	98.18	71.63	3.83	24.54
Granular Raney Ni	96.49	56.83	3.52	39.65
Raney Ni/PA	90.69	37.95	undetectable	62.05
